# Cholestasis and behavioral disorders 

**Published:** 2021

**Authors:** Delaram Eslimi Esfahani, Mohammad Reza Zarrindast

**Affiliations:** 1 *Department of Animal Biology, Faculty of Biological Sciences, Kharazmi University, Tehran, Iran*; 2 *Department of Neuroscience, School of Advanced Medical Technologies, Tehran University of Medical Sciences, Tehran, Iran *

**Keywords:** Acute liver failure, Cognitive impairments, Neurotransmitter systems

## Abstract

Acute and chronic failure in liver function may give rise to cognitive and non-cognitive impairments in the brain, namely hepatic encephalopathy (HE). Liver diseases may cause cholestasis, which is defined as the impaired secretion of bile. It is characterized by the accumulation of substances in plasma that are normally excreted in bile such as bile acids. Cholestasis can lead to hepatic encephalopathy. Several investigations have indicated that HE induces several symptoms, such as the impairment of learning and memory, anxiolytic-like behaviors, alterations in sleep pattern, and tremors. It has been reported that after HE, all classical neurotransmitter systems such as opioidergic, dopaminergic, cholinergic, GABAergic, adrenergic, serotonergic, and glutamatergic systems can be altered. This review focuses on cholestasis, hepatic encephalopathy, and behavioral disorders.

## Introduction

 Patients with liver diseases as well as animal models of chronic liver failure may show notable impairment in cognitive and non-cognitive functions (1). Acute or chronic liver failure may induce hepatic encephalopathy (HE) (2), which may present many different grades, from minimal HE to coma and death (3). Several investigations have indicated that HE induces several symptoms such as the impairment of learning and memory anxiolytic-like behaviors, alterations in sleep patterns, and tremors (3). It has been reported that after HE, all classical neurotransmitter systems such as opioidergic (4, 5), dopaminergic (6), histaminergic (7), cholinergic (8), GABAergic (3), adrenergic, serotonergic (9), and glutamatergic (10) systems can be altered.

Cholestasis, defined as the impaired secretion of bile, can be caused by liver diseases. It is described by various degrees of symptoms, mainly jaundice, pruritus, increased serum levels of alkaline phosphatase, GGT (γ-glutamyl transpeptidase), 5′-nucleotidase, bile acids, and cholesterol (11). Bile acid retention reduces new bile acid synthesis, which, in turn, results in decreased bile salt pool and dysregulation in the enterohepatic recirculation. Several experimental models have tried to elicit hepatic encephalopathy in lab animals (11). The two of the most commonly used models are carbon tetrachloride (CCl4) administration and common bile duct ligation (BDL) (11). CCl4, a compound that causes severe hepatic damage by inducing oxidative stress, is one of the most commonly used methods to elicit hepatic encephalopathy; however, it is considered to be an extremely toxic method, as it causes lipid peroxidation in liver parenchymal cells (12).

A marked elevation in endogenous opioid levels has been shown in both the plasma of patients with cholestatic liver diseases and animal models of cholestasis (4). Thus, it is suggested that endogenous opioids are implicated in the pathophysiology of cholestasis (5). Moreover, it has been documented that alterations in the release of the corticotrophin- releasing hormone (13, 14) and changes in manganese levels in the brain (15) are involved in altered cognitive and non-cognitive behaviors induced by cholestasis.

**Cholestasis and anxiety**

Some investigations have revealed that cholestasis decreases anxiety-like behaviors (16, 17). It has been elucidated that cholestasis alters the activity of all classic neurotransmitter systems such as opioidergic (18) and dopaminergic (6) systems.

Anxiety disorder is a psychiatric disorder characterized by somatic, cognitive, behavioral, and perceptual symptoms. Anxiety can be induced by many endocrine, autoimmune, metabolic, and toxic disorders as well as the adverse effects of medication (19).

Animal studies have shown that an opioid central pathway regulates bile secretion. Endogenous opioids are known to modulate cell growth. In cholestasis, the opioidergic system is hyperactive, and in cholangiocytes, a higher expression of opioid peptide messenger RNA has been seen (20).

The plasma levels of endogenous opioid peptides, mainly methionine enkephalin, have also been shown to increase in cholestatic patients and rats (5). There is evidence showing that opioids play a role in the pathophysiology and manifestations of cholestasis (21). 

Three classic types of opioid receptors (i.e. mu, delta, and kappa) belonging to the super-family of G-protein receptors are involved in major opioid actions, including anxiety, analgesia, reward, and the development of analgesic tolerance and physical dependence (22). Several studies have reported an anxiolytic function for morphine and mu-opiate receptor agonists when injected peripherally (23), as mu-opioid receptor antagonists tend to be anxiogenic (24). It has been suggested that the anxiolytic effect of opiates is mediated by their interaction with the GABAergic system in some specific brain areas such as the amygdala (25, 26).

Studies have shown the role of the opioidergic system in some cholestatic-induced behaviors. Functional interactions between the opioidergic system and cholestasis have also been demonstrated in the modulation of this behavior. Although the mechanism of cholestasis in anxiety-like behaviors has not been fully elaborated, it is interesting to note that anxiety-like behaviors have been reduced (16). In line with the current findings, the results of some other investigations have also shown an anxiolytic-related effect for morphine following systemic (23), intracerebroventricular (27), intra-ventral hippocampal or intra-nucleus accumbens (28) injection. Thus, the present results strongly support the involvement of the opioidergic system in anxiolytic-like behaviors induced by cholestasis.

Opiate antagonists have also been found to exhibit a reaction similar to that of opiate withdrawal in cholestatic patients who have not been previously treated with opiates (4). Down-regulation of mu-opioid receptors in the brain is also another behavior observed in cholestatic rats (5). Taken together, these observations strongly suggest that central opioidergic tone is increased in cholestasis syndrome, possibly due to the increased availability of endogenous opioid agonists at opioid receptors in the brain. The source of such increased levels of endogenous opioid peptides in cholestasis has not yet been established, but is likely to be in the cholestatic liver itself (21).

Several studies have indicated that the two major dopaminergic systems (i.e. mesolimbic and mesocortical) are involved in anxiogenic- and anxiolytic-like responses induced by some medications or acute stress (29). Based on the biochemical and pharmacological properties, two main subfamilies of dopamine receptors have been described. These include dopamine D1-like (D1 and D5) and dopamine D2-like (D2, D3, and D4) subfamilies (30).

Given the close link between opioidergic and dopaminergic systems with regard to anxiety-like behavior regulation (31), it is possible that opioidergic systems induce anxiolytic-like behaviors through interactions with dopaminergic systems in different brain sites. Such interactions seems to occur within mesolimbic projections. The activation of dopaminergic neurons of nucleus accumbens and ventral tegmental area in particular could be mediated by opioid peptides (32). Earlier studies have indicated dopaminergic system changes in cholestatic subjects as well as the involvement of dopaminergic systems in the regulation of anxiety-like behaviors.

The present results strongly suggest the involvement of the dopaminergic system (dopamine D1 and D2 receptors) in cholestasis-induced anxiolytic-like behaviors. They are completely in line with previous investigations that have shown that both dopamine D1 and D2 receptors are involved in morphine-induced anxiolytic-like behaviors (31). It has been shown that systemic injection of apomorphine decreases anxiety-like effects of the elevated plus-maze test via the dopamine D2 receptor subtype (33). However, our previous data indicated that the intra-ventral hippocampal injection of apomorphine induces anxiogenic-like effects (34) which may be due to the effect of apomorphine on the dopamine D2 presynaptic receptor and decreases in the dopamine level. It should be considered that in our previous experiments, dopamine receptor antagonists decreased anxiogenic-like effects when they were injected into the nucleus accumbens (35) and ventral hippocampus (34). However, these antagonists when injected peripherally reduced anxiolytic effects by bile duct ligation. It may be assumed that the antagonist might directly affect either the release or the turnover of morphine which induces anxiolytic-like activity. Moreover, some investigations have pointed out the role of dopaminergic systems in morphine-induced behaviors, namely the typical anxiolytic-like effects (16, 32, 31), memory impairments (36, 37), and reward (38). Therefore, it can also be inferred that opioids possibly exert their anxiolytic-like effects through dopamine release and dopaminergic system activation. 

On the other hand, cholestasis may alter the activity of different enzymatic pathways such as monoamine oxidase (MAOB). According to the literature, the hippocampal activity of this enzyme in astrocytes and radial glial cells (39) is decreased and increased two and six weeks after cholestasis, respectively (7). It has also been found that in thioacetamide-induced hepatic encephalopathy, the level of dopamine decreases in the frontal cortex and hippocampus (9). Moreover, in portosystemic drug-induced hepatic encephalopathy, the level of dopamine decreases in the striatum (40). All of this taken together, it may be concluded that the anxiolytic-like behaviors induced by bile duct ligation are possibly mediated through dopamine D1 and D2 receptor mechanisms.

The mesocortical and mesolimbic dopaminergic systems’ roles in anxiety-like behaviors (29, 34) have received as much attention as the GABAergic, serotonergic, and noradrenergic systems (41, 42). 

**Cholestasis-induced amnesia**

As previously indicated, BDL leads to biliary cirrhosis within 3–4 weeks (43), which occurs in conjunction with fibrosis, portal hypertension, portal-systemic shunting, and immune system dysfunction (43, 44). Cholestasis-induced HE would correspond to HE type C, which is associated with liver cirrhosis (32). Patients with liver cirrhosis without clinical symptoms of HE may show mild cognitive impairment (45). However, patients with manifestations of HE may present impairment of attention, memory, and cognitive function. 

On the basis of an earlier study, it has been clearly explicated that alterations in the activity of neurotransmitters, such as morphine and acetylcholine, and their interactions at both systemic and focal levels changed the formation of the memory process in the acquisition, consolidation, and retrieval levels in different parts of brain, particularly the dorsal hippocampus (46, 47). Thus, the following question is raised: Is the change in the activity of opioidergic and cholinergic systems following BDL involved in the impairment of memory formation induced by BDL?

It has been reported that the activity of acetylcholinesterase enzymes (AChE) was reduced in rat livers 3–4 weeks following BDL (48). It is also well known that the cholinergic receptor mechanism is involved in memory processes (49, 50) and acetylcholinesterase inhibitors improve memory. In contrast, anticholinergic drugs impair learning and memory in a variety of tests (46). It should also be considered that cholinergic activity modulates synaptic plasticity (51) and facilitates long-term potentiation (LTP) in various areas of the brain. Interestingly, the hippocampal formation is innervated by cholinergic fibers derived from the medial septum/vertical limb of the diagonal band of Broca (52), which is an important system for cognitive functions.

According to the results of previous studies, there is an interaction function between opioidergic and cholinergic systems in the CA1 of the dorsal hippocampus. Moreover, acute and chronic morphine administration increases hippocampal acetylcholine which may play a role in the ‘memory’ of the rewarding effect (53). The current data indicated that cholestasis impaired memory formation after bile duct ligation.

Some studies have shown an impaired ability to discriminate between the novel object and the pre-experience sample object in cholestatic rodents and spatial memory in the Morris water maze task after BDL (54). Moreover, deficits in attention and visual perception, ability impairment in performing memory tasks, and working memory in patients with HE via hippocampal formation have been reported, which may negatively regulate proliferation in adult hippocampal neurogenesis, eliciting memory impairments (55). Even if it is assumed that the deficits are cognitive (rather than sensory, perceptual, or motivational), it cannot be determined whether this is a learning deficit, a problem of acquisition, consolidation and storage, and/or a retrieval deficit. Nonetheless, the present study investigated the effects of opioidergic and cholinergic systems in the retrieval memory process in BDL mice.

The data showed that the pre-testing intra-CA1 administration of mecamylamine (a nicotinic receptor antagonist) and scopolamine (a muscarinic receptor antagonist) in the doses used decreased memory retrieval but did not alter anxiety-like behaviors (56). The data may indicate that muscarinic and nicotinic receptor systems of CA1 are involved in cholestasis-induced amnesia (57). To date, two nicotinic (nAChRs) and muscarinic (mAChRs) receptors have been identified (58). This is in agreement with suggestions that acetylcholine in hippocampal formation is an important neural substrate for cognitive function. The levels of ACh are continuously regulated by choline acetyltransferase, which synthesizes Ach, and by acetylcholinesterase, which rapidly degrades the neurotransmitter at cholinergic synapses, terminating synaptic transmission (59). A large number of studies have reported that muscarinic cholinergic agonists and acetylcholinesterase inhibitors, which enhance the availability of acetylcholine in the synaptic cleft, improved memory, while anticholinergic drugs impaired learning and memory in a variety of tasks, which further supports the present data (56). There is also experimental evidence that cholinergic activity modulates synaptic plasticity and facilitates long-term potentiation in various areas of the brain (60).

The present data indicated that the pre-testing co-administration of an ineffective dose of naloxone plus subthreshold doses of mecamylamine or scopolamine as well as ineffective doses of mecamylamine plus scopolamine reversed cholestasis-induced amnesia but did not alter anxiety-like behaviors (8). The results strongly indicated that the synergistic effects could influence opioidergic and cholinergic systems on the restoration of memory retrieval impairment induced by cholestasis (8). Opiate administration can cause an increase in acetylcholine levels in the whole brain. It has also been reported that only high doses of opiates elevate acetylcholine levels in the hippocampus (61).

**Effects of cholestasis on rewarding and exploratory behaviors**

A psychological reward is fundamental to the organization of behavior, which induces pleasure and supports elementary processes such as drinking, eating, and reproduction. The cortical-basal ganglia circuit is highly involved in the reward system, though cells in many other brain regions may also respond to reward (62). The anterior cingulate cortex, orbital prefrontal cortex, ventral striatum, ventral pallidum, and midbrain dopamine (DA) neurons are main structures in the reward network. The neurotransmitter DA has also been shown to play an important role in the reward phenomenon (36, 63). Five different DA receptors have been identified which are G protein-coupled and categorized as belonging to one of the two classes designated as D1-like (D1 and D5) or D2-like (D2, D3, and D4) (30). Autoreceptors, which are D2-like, have been identified on the presynaptic terminals of dopaminergic cells. D1-like receptors, however, can stimulate adenylyl cyclase activity and increase cyclic adenosine monophosphate (cAMP). Conversely, D2-like receptor activation either inhibits or has no effect on cAMP levels (30-). 

Opiates elicit rewarding effects at the level of the mesolimbic DA system that originates from the ventral tegmental area (VTA) and projects to the nucleus accumbens (Nac). A large body of evidence has demonstrated that the activation of VTA DA neurons via inhibition of GABAergic inhibitory interneurons causes an increase in DA neurotransmission to the Nac and induces a morphine reward (64). Observations indicating the increase in endogenous opioids are compatible with a global down-regulation of mu-opioid receptors in the brains of BDL rats (5). Opiates and endogenous opioids have attracted increased research interest, because opioids produce a psychologically reinforcing effect which can result in their abuse. It is believed that the conditioned place preference (CPP) paradigm creates a preference for a context due to the contiguous association between the context and the drug stimulus. It can be used as a model for studying the reinforcing effects of drugs with dependent liability (65). 

Researchers have stated that the expression and secretion of serotonin, endogenous opioid peptides, and neurotrophins as well as their corresponding receptors increase during cholestatic diseases (4). In cholestasis, the liver is not the only source of met-enkephalin, although it alters expression of the delta opioid receptor to which met-enkephalin preferentially binds (66). Thus, one can propose that met-enkephalin has a local function in the cholestatic liver. Although the reason for alteration in the number of opioid receptors in cholestasis is not yet fully understood, it has been shown that an increase in the availability of opioid peptides in the periphery may help their alteration into the central nervous system (4).

The present data showed that the conditioning treatments with different doses of morphine produced a dose-related place preference in sham-operated mice (67). The data was consistent with those of previous reports, which have suggested that the conditioning procedure could be used to investigate the reward effect of morphine (63, 21, 67). While the intermediate (medium) dose of morphine produced CPP in sham operated mice, a higher dose of the opioid was required in BDL animals to elicit CPP. It could thus be concluded that the morphine dose response curve has shifted to the right in BDL animals, which could be in line with the findings that mu-receptor levels are down-regulated in BDL animals (67). Thus, the middle dose of morphine, which was an effective dose in sham-operated animals, could be ineffective in the BDL group. In addition, previous studies have suggested that elevation of endogenous opioids may induce nitric oxide (NO) overproduction in cholestatic rats. The endogenous NO could play a role in the modulation of dopaminergic effects elicited by morphine. The nucleus accumbens is one region where NO is implicated in the control of DA release (67).

In agreement with our previous studies, the self-administration of naloxone-induced CPP in mice is an effect mediated by the central nervous system (46). BDL also reduced the CPP response that was induced in sham-operated animals. A small right-shift was observed in the aversive effects of naloxone (intermediate dose in sham versus higher dose in BDL animals), which could be related to down-regulated opioid receptors in the brain. Naloxone would compete with local met-enkephalin for binding to the delta opioid receptor expressed by proliferating bile ducts (66). 

The main DA receptor subtypes (D1 and D2) have been proposed to play a critical role in the incentive aspect of opiate reward. Activation of these receptors could be essential for the development of addiction to opiates. In addition, DA D1-like receptors may play a critical role in reward-related learning. Possibly, rewarding stimuli such as morphine may produce this type of learning (68, 69). DA also has an essential role in associative stimulus-reward learning. Other investigations have shown that either place preferences or place aversions can be induced by D2 receptor activation. Previous studies observed no effect on place conditioning for DA antagonists, which preferentially act at DA D2 receptors (70). 

**Impaired passive avoidance performance induced by cholestasis**

The hippocampal formation plays a key role in learning and memory processes, including acquisition, consolidation, and retrieval functions of various types of memories (71, 72, 73). This site has important neurotransmitter systems such as DA and opioids which are known to be central in learning and memory mechanisms (71, 72). The hippocampus receives DAergic afferents (29, 72) involved in cognitive and non-cognitive behavioral functions (29, 71, 74). 

Several reports have shown that the long-term potentiation (LTP) in the dorsal part of the hippocampus (CA1) strongly depends on DA (72). Our previous studies have postulated that there seems to be a close relationship between opioidergic and DAergic systems in various functional responses in the brain (69, 71). Referring to previous studies and having known the altered opioidergic and DAergic systems in patients with hepatic encephalopathy could potentially modulate learning and memory.

The working memory associated with the function of hippocampal formation is reported to be impaired in patients with HE (75). Memory impairments have also been found in animal models of chronic liver failure. BDL rats are shown to suffer markedly diminished learning ability in the Morris water maze task (54). Moreover, it has been previously postulated that the TNF alpha receptors’ mRNA expression is elevated in the hippocampus of BDL mice. Upregulation of this pro-inflammatory cytokine receptor is shown to negatively affect progenitor proliferation in the adult brain’s hippocampal neurogenesis, leading to memory impairment (76, 77). It is well known that the reduction in hippocampal long-term potentiation can be responsible for memory impairment in HE. On the other hand, after cholestasis, the HE-induced working memory impairment may have been indirectly caused by hyperammonemia (78, 79). 

Reports have shown that HE alters the activity of opioidergic and DAergic systems (78). These systems play a critical role in learning and memory formation in the CA1 region of the hippocampus (80, 81) and, thus, may similarly be involved in BDL-induced amnesia. Given the three phases of the memory formation process, i.e. acquisition, consolidation, and retrieval, it is not yet fully understood whether this phenomenon is related to deficits in memory acquisition, consolidation, and/or retrieval. 

The present data indicated that sole pre-test intra-CA1 administration of naloxone does not alter memory performance in passive avoidance and exploratory behaviors; however, it reversed BDL-induced amnesia with no effect on anxiety-like behaviors (82). This may indicate that, following BDL-induced HE, the CA1 opioid receptor mechanism is activated to induce amnesia. It is also likely that the elevation in plasma concentrations of endogenous opioids mediates the syndromes associated with HE (83). Moreover, intracerebroventricular administration of small amounts of opioid agonists leaves deleterious effects on hepatocytes (18). Opioid receptor antagonist administration in patients who experience HE-related pruritus has led to alleviation of their symptoms without inducing significant changes in the degree of HE (83). 

The involvement of the mu-opioid receptors in acute and chronic effects of opioid compounds is well described. The activation of mu-opioid receptors alters intracellular signaling proteins, including inwardly rectifying potassium channels, calcium channels, phospholipase C as well as the mitogen-activated protein kinase (MAPK) pathways (84). Earlier investigations have clarified that the deactivation of mu-, delta-, but not kappa-opioid receptors by their antagonists improves memory retrieval through separation of the biological functions associated with mu-, delta-, and kappa-opioid receptors. Therefore, activation of mu and delta receptor induces positive reinforcement, while selective kappa agonists produce aversive responses (85). Similar effects have been observed in the locomotor activity, conditioned place preference, and intracranial self-stimulation phenomena. The opposite reaction has been shown to occur on the membrane level, i.e. mu and delta selective agonists open the special type of potassium channels, while kappa agonists control calcium permeability (86).

Both substantia nigra (nigrostriatal system) and ventral tegmental areas (mesolimbic system) extend DAergic projections to the hippocampus. These pathways are involved in the modulation of anxiety and memory (87). In addition, it has been shown that DA metabolizing enzymes such as COMT or DA uptake sites are also present in the hippocampus. HE may alter the activity of enzymes such as monoamine oxidase (MAO B) through different mechanisms. In thioacetamide-induced HE, the DA level of the frontal cortex and hippocampus decline, while in drug-induced porto-systemic HE, the striatal DA level decreases instead (7).

The present data demonstrated that intra-CA1 administration of sulpiride (a dopamine D2-like receptor antagonist), but not SCH23390 (a dopamine D1-like receptor antagonist), reverses the BDL-induced amnesia, while it does not alter other exploratory behaviors (88). Although the higher dose of SCH23390 was shown to improve HE-induced amnesia, this response cannot be considered reliable, as the intervention negatively affected locomotor activities. In addition, sole administration of the higher dose of SCH23390 induced anxiogenic-like behaviors, suggesting that under normal circumstances, the dorsal hippocampal DAergic system potentially exerts a physiological influence on anxiety-like behaviors through DA D1-like receptors (78). Based on insights from biochemical studies, dopamine modulates adenylyl cyclase activity via two receptor family groups, i.e. DA D1-like and D2-like receptors, which are closely related to G protein-coupled receptors (89). 

Activation of DA D1-like receptors increases adenylate cyclase activity and thereby the intracellular production of cAMP. In addition, activation of DA D2-like receptors either decreases or elicits no effect on cAMP synthesis through inhibition of guanine nucleotide proteins (Gi) (90). It has been made clear that deactivation of DA D1-like but not D2-like receptors disrupts memory retrieval. Moreover, the co-administration of the subthreshold dose of naloxone with the subthreshold dose of SCH23390, but not the subthreshold dose of sulpiride, reverses BDL-induced memory impairment while exerting no effect on other exploratory behaviors. Co-administration of similar doses of SCH23390 with sulpiride also reversed BDL-induced amnesia (88). 

The outcomes of earlier investigations seem to support the present data. For example, referring to the anatomical evidence, opiate-containing terminals are known to be distributed in close association with DAergic neurons (91 This may additionally suggest that DAergic and opioidergic systems are likely linked in the endogenous modulation of memory retrieval (92). The response induced by the combination of SCH23390 and naloxone is possibly due to the relatively high affinity of SCH23390 toward serotonergic receptors (93). It has been shown that mu- and delta-opioid receptors increased while kappa opioid receptors decreased synaptic serotonin levels, which may explain the synergic response caused by naloxone and SCH23390 (94). 

**Long-term memory in bile duct ligation rats**

Omitted Sentences. The main findings of the authors’ studies indicate that memory retrieval (short-term memory) is impaired 7 days after BDL, and it worsens with the progression of cholestasis 21 days after bile duct ligation in rats (95). Moreover, no alterations were seen in early stages of the disease, but impairment was observed 21 days post-BDL. The authors’ previous data also showed that short-term memory was not altered in the early stage of cholestasis in BDL rats (95). Mild cognitive impairment has been reported in patients with liver cirrhosis (96). Furthermore, in some patients with liver disease and signs of hyperammonemia, impaired attention, memory, cognitive function, and motor function have been observed. The current findings are consistent with some studies that have reported impaired spatial memory and ability to discriminate the novel object after BDL in rodents (88). Of course, the onset time of disturbances has not been studied. Moreover, in patients with liver disease, deficits in attention, visual perception, and working memory have been reported (13).

The molecular mechanisms by which liver failure impairs cognitive function remain unknown. Some studies suggest that in liver disease, hyperammonemia is one of the main factors responsible for neurological alterations (97). It has also been suggested that some mechanisms for the glutamatergic system are involved in induced amnesia, such as change of brain NO, oxidative stress, disruption of calcium homeostasis, membrane damage, and cell death (98). All of the above-mentioned biologic effects can result in cognitive deficits in amnesia induced in BDL rats. Nevertheless, the mechanisms of amnesia that were induced by cholestasis in BDL rats have not been fully elaborated.

**Cholestasis impaired spatial and non-spatial novelty detection**

Patients with manifestations of HE may present with impairments of attention, memory, learning, and cognitive function as well as some alterations in motor function, including psychomotor slowing, bradykinesia or hypokinesia, and asterixis (99). These behaviors in patients with cholestatic liver disease are of a central and not a peripheral origin. Some studies have shown an impaired ability to discriminate between the novel and the previously encountered sample object as well as impaired spatial memory acquisition in the Morris water maze task two to three weeks after BDL in cholestatic rodents (54). Other reports suggest deficits in visuo-spatial abilities and working memory in patients with hepatic encephalopathy (HE) which are possibly due to changes in the hippocampal formation (96, 100).Yet other investigators have suggested that deficits in the release of corticotrophin-releasing hormone (14) are involved in cholestasis-induced behaviors. Given the three phases of the memory formation process, i.e. acquisition, consolidation, and retrieval, it is not yet fully understood whether this phenomenon is related to deficits in memory acquisition, consolidation, and/or retrieval. The present data indicated that cholestasis impaired both spatial and non-spatial memory formation (73).

**Cholestasis-induced hypothermia**

The hypothalamic preoptic area (POA) plays a major role in thermoregulation of the body (101). This structure sends efferent signals to various effector organs. It also senses local brain temperature and has thermo-sensitive neurons which change their activity in accordance with the local brain temperature. The hypothalamic nuclei are heavily influenced by inputs from other areas of the brain. They receive projections from the hippocampus (CA1), amygdala, and prefrontal cortex. It is well known that the hypothalamic regions near preoptic (a thermoregulatory site), supraoptic, and paraventricular nuclei receive direct inputs from the CA1ventral field (102, 103, 104

The hippocampus receives dopaminergic afferents both from substantia nigra and VTA. Both dopamine D1 and D2 receptor families are found in the hippocampus (29). Moreover, much of the dopaminergic transmission-associated enzymatic machinery, including dopamine uptake sites, dopamine metabolizing enzymes, and COMT, are abundantly present in the hippocampus. The dopamine receptor system has been implicated in the regulation of the central body temperature (105). Dopamine receptor activation has been reported to produce hypothermia in several species. There are also experiments showing the effects of specific dopamine receptor blockers on core and skin temperatures in rats under different stress conditions. Based on the current evidence, dopamine, at least in rodents, plays a physiological role in central thermoregulatory mechanisms (106).

The present data showed that compared to the non-operated control groups, BDL reduces the body temperature two and four days after it is done. The results are in agreement with other investigations, showing that BDL reduces body tmperature one and three days after BDL in rats (107). The mechanism(s) of BDL-induced hypothermia is (are) have not been made fully clear to date; however, the following may be some possible mechanisms involved in this phenomenon.

Morphine can produce hypothermia or hyperthermia depending upon dose, species, methodologies, and the ambient temperature. Most reports show that low to moderate doses induce hyperthermia, whereas hypothermia occurs at higher doses (107). Several studies have also shown that various brain physiological and behavioral functions are influenced by the opioidergic system. Therefore, in the present study, the cholestasis-induced increase in endogenous opioids might have possibly contributed to hypothermia. 

Morphine is shown to result in dopamine release in several regions of the brain, such as the hippocampus. Thus, cholestasis may affect thermoregulation through increased serum morphine levels (17) and thus dopamine level alterations in different brain nuclei such as the hippocampus. The distribution of the dopaminergic system appears to be particularly strong in the subiculum, hilus, and CA1 regions, and both dopamine D1 and D2 receptor families are found in the hippocampus (108).

It has been reported that distinct consequences of cholestasis, such as HE, increase the activity of monoamine oxidase in the brain. There is also a report indicating an increase in hippocampal and frontal cortical dopamine in a thioacetamide model of hepatic encephalopathy (109). 

In the current study, intra-CA1 injection of SCH23390 (SCH) and sulpiride (SUL) did not alter body temperature individually. Thus, it may be concluded that the CA1 dopaminergic system does not influence the body thermoregulatory mechanism(s) (110). This is inferred from the observation that the administration of dopamine antagonists is not associated with alterations in body temperature. On the other hand, while intra-CA1 administration SCH could not alter BDL-induced hypothermia, SUL partly reversed this phenomenon, indicating that CA1 D2, but not dopamine D1, receptors are potentially involved in BDL-induced hypothermia. This result is in agreement with other investigations showing that dopamine D2 receptors are mainly involved in body thermoregulation (111). Moreover, sole intra-CA1 injection of both SKF38393 (SKF) and quinpirole (QUI) did not alter body temperature, while these drugs partly reversed BDL-induced hypothermia (110). The findings indicate the specific and independent roles of dopamine receptors (D1 and D2) in thermoregulation. In addition, the response seen with these drugs may depend on how presynaptic and postsynaptic receptors are affected. For example, SUL affects the presynaptic receptors such that dopamine levels in the synaptic cleft are increased. Similar effects on BDL-induced hypothermia are seen with QUI and SKF (110).

Tubero-infundibular neurons of the preoptic/anterior hypothalamus (PO/AH) and mesolimbic and nigrostriatal dopaminergic pathways have also been implicated in body thermoregulation (112). However, norepinephrine and 5-hydroxytryptamine (5-HT) are the two principal neurotransmitters in the hypothalamus which functionally modulate heat loss and heat gain mechanisms, respectively (101). Dopamine is implicated in the central control mechanism of temperature regulation. Classically, the tubero-infundibular region of the hypothalamus, the mesolimbic region, and the nigrostriatal pathways are identified as regions containing high concentrations of dopaminergic neurons (101).

Some studies have revealed that peripheral and central administration of apomorphine (D1/D2 dopamine receptor agonist) induced hyperthermia in rodents. It has been suggested that the response of dopamine D1 receptor agonists is elicited by a central mechanism. Conversely, there are investigations substantiating that dopamine D1 receptor stimulation has no effect on temperature (113). These discrepancies could be explained by differences in experimental animal species and the drugs which were used in different experiment setups.

There exists suggestions that the hypothermia induced by dopamine D2 receptor activation can be inhibited by dopamine D1 receptor stimulation. In contrast, the hyperthermia induced by dopamine D1 receptor stimulation can be inhibited by dopamine D2 receptor activation (114). Therefore, it can be inferred that the two receptor subtypes possibly act in the opposite direction. This may be explained by the opposite regulation of adenylate cyclase activity by dopamine D1 and D2 agonists, which was reported years ago (114,115).

Hypothermia has also been induced when apomorphine was injected into the nucleus accumbens, caudate, and PO/AH (116). It is also noteworthy that the apomorphine response is antagonized by bilateral lesions in the caudate nuclei and that D-amphetamine hypothermia is inhibited following unilateral lesions in the mesolimbic system (116). The data in the present study revealed that A) cholestasis may induce hypothermia, and B), the CA1 dopaminergic system is possibly involved in this phenomenon.

## Conclusion

In conclusion, this review showed that cholestasis leads to developed cognitive impairments such as amnesia and memory disorders, anxiety, rewarding and exploratory behaviors and hypothermia with progression of liver failure in BDL rats.
